# First-in-man study of coronary stent 3D reconstruction in the cathlab using rotational angiography

**DOI:** 10.1093/ehjimp/qyaf065

**Published:** 2025-05-28

**Authors:** Hakim Benamer, Liliane Ramus, Hachem-Ali Haidar, Matthieu Perier, Mehdi Saighi Bouaouina, Sophie Amelot, Régis Vaillant

**Affiliations:** Department of Cardiology, ICV GVM La Roseraie, 93300 Aubervilliers, France; Department of Cardiology, Foch Hospital, 92150 Suresnes, France; Department of Cardiology, Cardiovascular Institute Paris Sud (ICPS), 91300 Massy, France; GE HealthCare, Interventional Image Guided Systems, 283 rue de la minière, 78530 Buc, France; Department of Cardiology, ICV GVM La Roseraie, 93300 Aubervilliers, France; Department of Cardiology, Foch Hospital, 92150 Suresnes, France; Department of Cardiology, ICV GVM La Roseraie, 93300 Aubervilliers, France; Department of Cardiology, Foch Hospital, 92150 Suresnes, France; Department of Cardiology, ICV GVM La Roseraie, 93300 Aubervilliers, France; GE HealthCare, Interventional Image Guided Systems, 283 rue de la minière, 78530 Buc, France; GE HealthCare, Interventional Image Guided Systems, 283 rue de la minière, 78530 Buc, France

**Keywords:** coronary artery disease, coronary stenting, stent expansion, stent assessment, stent imaging, stent enhancement

## Abstract

**Aims:**

Stent under-expansion is a well-known predictor of post-percutaneous coronary intervention (PCI) major adverse cardiovascular events (MACE). This article presents a new technique to image coronary stents in 3D in the cathlab utilizing only the angiographic equipment.

**Methods and results:**

Thirty patients with an indication of PCI were consented and prospectively included. They underwent a 3D rotational angiography following stent deployment without contrast injection. The 3D reconstructions of the coronary stents were independently reviewed offline by three experienced interventional cardiologists. Image quality was assessed using a qualitative scale: Excellent (struts are highly contrasted and sharp), Good (struts are contrasted enough to be distinguished from the background), Fair (the overall shape of the stent can be assessed but the precise demarcation between struts and background may be ambiguous in some areas) or Limited (it is challenging to distinguish the struts from the background). The 3D stent reconstruction quality was evaluated as Excellent for 37.8% of the cases, Good for 33.3%, Fair for 22.2%, and Limited for 6.7%. Substantial to near-perfect agreement was observed among reviewers (weighted Cohen’s kappa 0.8, 0.78, 0.82 for the three pairs of reviewers respectively). The 3D quality was significantly correlated with the BMI using the Spearman rank correlation coefficient (*ρ*=0.74, *P*-value < 0.001).

**Conclusion:**

The study demonstrates that 3D rotational angiography is feasible during PCI. The 3D reconstructions were evaluated as excellent or good in most patients by three interventional cardiologists. Additional studies are necessary to validate the measurements with respect to intra-vascular imaging and evaluate the role of this new technique in stent optimization.

## Graphical Abstract

**Graphical Abstract:**
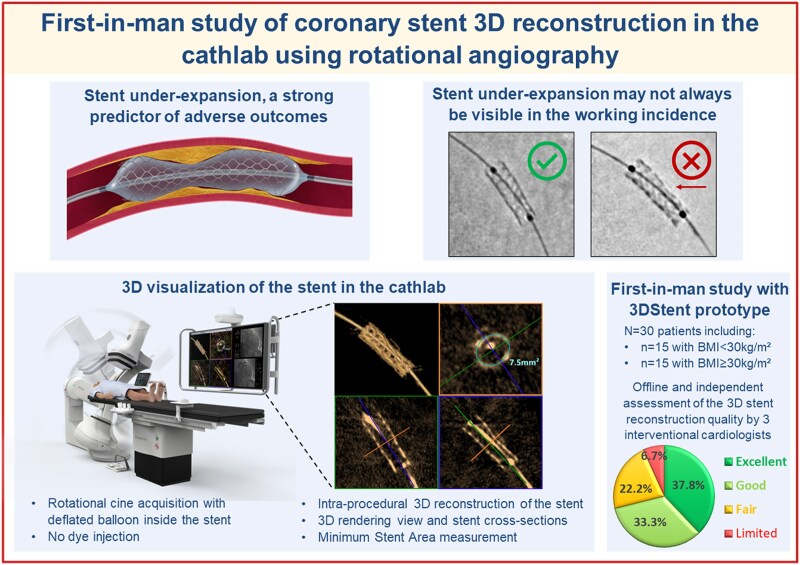

## Introduction

Guidance and assessment of stent delivery rely mostly on X-ray imaging and the availability of digital stent enhancement techniques^1,2^ has facilitated the procedure. It is widely used to assess intra-procedure stent deployment. Common practice is to perform a post-dilatation if the appearance of the deployed stent is visually judged sub-optimal.

The procedure is also commonly guided by intra-vascular imaging. Since this imaging modality emerged in the 1990’s, it has progressively helped interventional cardiologists learn how to optimally select the stent for a given pathology and how to deploy it. One of the key learnings is the importance of using high-pressure non-compliant balloons for post-dilation. In a retrospective analysis of several IVUS trials, Lee *et al*.^3^ found out that optimal stent expansion is associated with improved long-term clinical outcomes. Optimal stent expansion is defined as minimum stent area (MSA) > 5.5 mm^2^, > 5.0 mm^2^, or MSA/distal reference lumen area >90%.

Räber *et al*.^4^ established that stent under-expansion is a major predictor of stent failure. Stent expansion is usually described as minimum stent cross-sectional area either as an absolute measure (absolute expansion), or compared with the pre-defined reference area, which can be the proximal, distal, largest, or average reference area (relative expansion). Alfonso *et al*.^5^ found that in-stent restenosis (ISR) remains the most common cause of stent failure. ISR is characterized by a significant reduction in the luminal diameter within the stented segment after a successful percutaneous coronary intervention (PCI). In the USA, recent data^5^ has suggested that treatment of ISR may account for up to 10% of all PCI procedures performed, whereas in Europe it has been reported to constitute ∼5% of all PCI procedures.

These different studies have established the importance of assessing stent expansion and therefore the necessity of the technologies mentioned above. Stent enhancement is now considered a very common tool and has the advantage of being quick and easy both in terms of workflow and reading. However, stent enhancement remains limited, as it only provides a 2D view. Intra-vascular imaging remains the gold standard for assessing stent expansion as it provides cross-sections of the stent, but it is largely under-used due to cost reasons, and it also has the disadvantage of being more invasive. Being able to perform this 3D assessment in the cathlab without any additional device is an unmet need. In the work presented here, we introduce C-arm motion compensated computed tomography (CMCT) as a means to address this unmet need. In the following sections, we will discuss this technology from both practical aspects—how to acquire the images required by CMCT—and the principles behind this technology. After characterization of this methodology, a clinical study has been done (ANSM registration no. ID-RCB #2017-A00983-50) to collect 30 clinical cases. The objective of the study is to assess the applicability of the technology in the cathlab and demonstrate that the images can be interpreted by experienced cardiologists vs. clinical needs. In the absence of systematic use of intra-vascular imaging, it is not designed for validation against these gold standards. In this article, we describe the methodology followed and the obtained results.

## Methods

### Technical principle

#### Acquisition workflow

**Figure 1:**
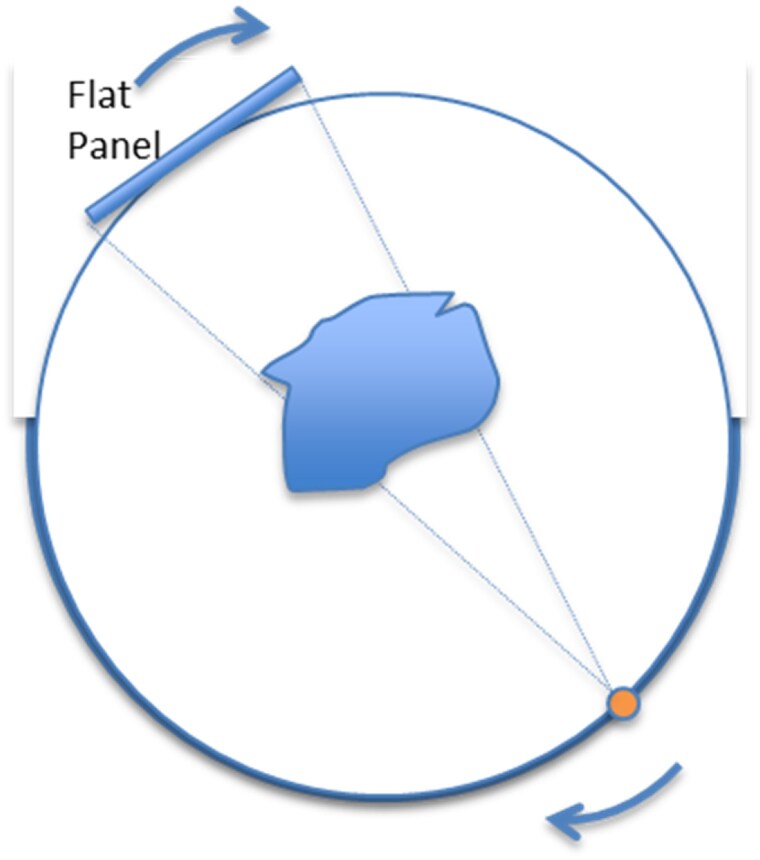
Imaging geometry: the gantry rotates over 200° from RAO 100° to LAO 100°. RAO, right anterior oblique; LAO, left anterior oblique.

3D rotational angiography of coronary stents is performed with a C-arm interventional system and does not require any additional device. The acquisition workflow is very similar to that of Cone Beam Computed Tomography (CBCT), which has been employed by interventional neuroradiologists since the end of the 90’s. It is an automated rotational acquisition covering 200° from RAO 100° to LAO 100° (see *Figure 1*). This acquisition is preceded by a step to centre the anatomy of interest. Centering is achieved by panning the table in the frontal position and adjusting the table height in the lateral position to ensure the stent remains in the field of view during the rotation. The collimator blades can be adjusted to limit irradiation to the useful area. The next step is an automated rotation at low speed to ensure no collisions occur with the patient, or any equipment in the room. During the test rotation there is no X-ray exposure. Once completed, the automated X-ray acquisition is started at the operator’s convenience. Exposure is automatically started and stopped as the gantry rotates. For imaging coronary stents, the rotational acquisition needs to be performed with a deflated balloon still in place inside the stent. This can be either the original stent delivery balloon or a post-dilation balloon. As explained below, the two markers of the balloon play a key role in the CMCT technology. An example of 3D rotational angiography is available in Supplementary data online, *Movie S1*.

#### CMCT technical principle

Computed tomography (CT) is a 3D reconstruction of patient anatomy with a tomodensitometry scanner starting from an X-ray based acquisition around the patient. With CBCT, this technology has been introduced to C-arm systems using a rotational acquisition covering 200°. The reconstruction itself is based on the retro-projection algorithm proposed by Feldkamp *et al*.^6^ It requires precise knowledge of the location of the focal spot and the flat panel, which is determined by calibration.

A major constraint for the clinical use of the techniques detailed above is the requirement to have static anatomy during acquisition. This constraint is managed by very high-speed rotation for CT scanners and was originally a barrier for 3D cardiac imaging on C-arms.

CMCT is a significant enhancement of the CBCT for the 3D imaging of moving anatomy or devices. Anatomic motion is determined by tracking landmarks in the projections. This motion is then used to adjust the calibration of the C-arm before applying the retro-projection algorithm.

For coronary stent 3D reconstruction, the retained landmarks are the markers of a deflated balloon positioned inside the stent. The position of the stent in the different projections is determined and not extrapolated based on the cardiac cycle. Thus, from a theoretical standpoint, this approach is robust to irregular heartbeats and respiratory motion, eliminating the need for rapid pacing.

The algorithm produces a volume of data with a voxel size of 0.1 mm. It can be visualized with different standard rendering methods either as a 3D volume with maximum intensity projection or volume rendering (VR) view. In an automated way, its display can be presented in multi-planar reformat (MPR) view aligned with the axis defined by the two markers providing an axial view perpendicular to this axis or a longitudinal view when this axis is included in the slice. Measurements are possible in these different views allowing for the determination of the stent diameters at any position or orientation in the axial view. Stent area at different levels of the stent can also be measured.

### First-in-man study: study design

From November 2017 to November 2019, 30 patients with an indication for PCI with drug-eluting stents (DES) implant were consented and prospectively included in the study. The study protocol was approved by the French Agency for the Safety of Health Products (ANSM registration no. ID RCB 2017-A00983-50) and received a favourable opinion from a French ethics committee (CPP Ile-de-France II). The patients underwent rotational angiography following stent deployment with an Innova IGS 520 system from GE HealthCare equipped with a prototype version of 3DStent, a recently introduced technique from GE HealthCare. Other steps of the procedure workflow were performed as standard care for this facility. Intra-vascular imaging is used only in some complex PCI cases, occurring in a single case in this study.

For each patient, the 3D acquisition was reconstructed with CMCT technology and the 3D reconstruction was reviewed offline and independently by three experienced interventional cardiologists (H.B., H.A.H., and M.P.). The image quality of the 3D reconstruction was assessed using a qualitative scale: Excellent (struts are highly contrasted and sharp), Good (struts are contrasted enough to be distinguished from the background), Fair (the overall shape of the stent can be assessed but the precise demarcation between struts and background may be ambiguous in some areas), or Limited (it is challenging to distinguish the struts from the background).

When a 2D stent enhancement (StentViz, GE HealthCare) sequence was also acquired following stent deployment, with no post-dilation done between the StentViz and the 3D acquisition, it was reviewed prior to the prototype 3D reconstruction. The relative clinical interest of both tools was assessed on a 3-level scale: 3D superior, equivalent, or inferior to StentViz.

### Statistical analysis

Inter-observer agreement was assessed using weighted Cohen’s kappa for each pair of reviewers. This index is a well-known statistical metric to quantify the agreement among two observers evaluating data samples among ordinal categories. For this computation, quadratic weights were used to account for the degree of disagreement (e.g. a disagreement between Fair and Excellent is more significant than between Good and Excellent). The weighted Cohen’s kappa ranges from 0 to 1, with 0 indicating no agreement and 1 indicating perfect agreement. Intermediate values in the ranges]0;0.2], ]0.2;0.4], ]0.4;0.6], ]0.6;0.8], and [0.8;1] indicate slight, fair, moderate, substantial, near-perfect agreements, respectively.

The Spearman rank correlation coefficient was used to assess whether the quality of the 3D stent reconstruction was significantly correlated with BMI and stent orientation on this dataset. This non-parametric measure allows to quantify the monotonicity of the relationship between two variables based on the ranks; it has the advantage of being robust to non-normal distributions, small datasets, and non-linear relationships between variables. It ranges from −1 to 1, with −1 (respectively 1) indicating a perfect negative (respectively positive) monotonic relationship between the variables, and 0 indicating an absence of monotonic relationship between the variables. For this calculation, each level of the image quality scale was assigned a numerical score: 1 for Excellent, 2 for Good, 3 for Fair, and 4 for Limited. The 30 samples could then be ranked according to a mean image quality score, calculated as the average of the scores given by the three raters. This strategy is only valid if the agreement between the three reviewers has reached a sufficient level. Finally, a two-tailed *P*-value ≤ 0.05 was considered statistically significant for this correlation result.

## Results

### Patient population

Patient characteristics are summarized in *Table 1*, lesion characteristics in *Table 2*, and procedural characteristics in *Table 3*.

**Table 1:** Patient characteristics for the cohort of 30 patients

Age, median value	67.5
Male gender, *n* (%)	73.3%
BMI (kg/m²), median value	29.7
Hypertension, *n* (%)	76.7%
Diabetes, *n* (%)	36.7%
Insulin-dependent diabetes, *n* (%)	0.0%
Dyslipidaemia, *n* (%)	46.7%
Family history of CVD, *n* (%)	13.3%
Smoking history, *n* (%)	33.3%
Clearance (mL/min), median value	91.5
Renal insufficiency, *n* (%)	0.0%
Prior MI, *n* (%)	13.3%
Prior CABG, *n* (%)	0.0%
Prior PCI, *n* (%)	26.7%
PCI indication	
Chronic coronary syndrome, *n* (%)	100.0%
Stable angina, *n* (%)	86.7%
Silent ischaemia, *n* (%)	13.3%
Acute coronary syndrome, *n* (%)	0.0%

BMI, body mass index; CVD, cardiovascular disease; MI, myocardial infarction; CABG, coronary artery bypass graft; PCI, percutaneous coronary intervention.

**Table 2:** Lesion characteristics for the cohort of 30 patients (lesion for which the stent was imaged with 3DStent prototype)

Target vessel	
Left main, *n* (%)	3.3%
LAD, *n* (%)	40.0%
LCx/obtuse marginal, *n* (%)	36.7%
RCA, *n* (%)	20.0%
Target segment	
Proximal, *n* (%)	23.3%
Mid, *n* (%)	56.7%
Proximal + Mid, *n* (%)	16.7%
Distal, *n* (%)	3.3%
Lesion type	
De novo, *n* (%)	100.0%
In-stent restenosis, *n* (%)	0.0%
ACC/AHA lesion type	
A, *n* (%)	3.3%
B1, *n* (%)	43.3%
B2, *n* (%)	16.7%
C, *n* (%)	36.7%
Calcification	
Null or minimal calcification, *n* (%)	73.3%
Moderate or severe calcification, *n* (%)	26.7%
Bifurcation lesion, *n* (%)	26.7%
Total occlusion, *n* (%)	0.0%
Lesion length (mm), median value	18.0

LAD, left anterior descending artery; LCx, left circumflex artery; ACC, American College of Cardiology; AHA, American Heart Association; RCA, right coronary artery.

**Table 3:** Procedural characteristics for the cohort of 30 patients

General procedural characteristics	
Access site	
Radial, *n* (%)	96.7%
Femoral, *n* (%)	3.3%
Guide catheter	
6F, *n* (%)	100.0%
7F, *n* (%)	0.0%
Number of lesions treated per patient, mean	1.40
Number of lesions treated per patient, SD	0.56
Number of stents used per patient, mean	1.63
Number of stents used per patient, SD	0.67
Treated length (total stent length), median value	30.5
Treated length (total stent length)≥ 60 mm, *n* (%)	13.3%
Overlapping stents, *n* (%)	16.7%
Total exam CAK (mGy), median value	681
Total exam DAP (Gy.cm²), median value	41.5
Fluoroscopy time (min), median value	6.4
Contrast (mLs), median value	80.0
Procedural characteristics related to the lesion/stent(s) imaged with 3DStent prototype	
Use of rotablation, *n* (%)	3.3%
Use of intra-vascular imaging, *n*(%)	3.3%
Pre-stenting non-compliant balloon dilation, *n* (%)	26.7%
Post-stenting non-compliant balloon dilation, *n* (%)	43.3%
Stent length (total stent length in case of overlapping stents), median value (mm)	21.0
Stent(s) type	
DES Xience, *n* (%)	46.7%
DES Firehawk Microport, *n* (%)	6.7%
DES Ultimaster, *n* (%)	26.7%
DES Biomatrix, *n* (%)	13.3%
DES Resolute Onyx, *n* (%)	3.3%
DES Biofreedom, *n* (%)	3.3%
3D acquisition CAK (mGy), median value	167.5
3D acquisition measured DAP (Gy.cm²), median value	16.6
Estimated 3D acquisition DAP (Gy.cm²) with reasonable top-to-bottom collimation, median value	6.3

CAK, cumulated air kerma; DAP, dose-area product; DES, drug-eluting stent.

### Visual results

**Figure 2:**
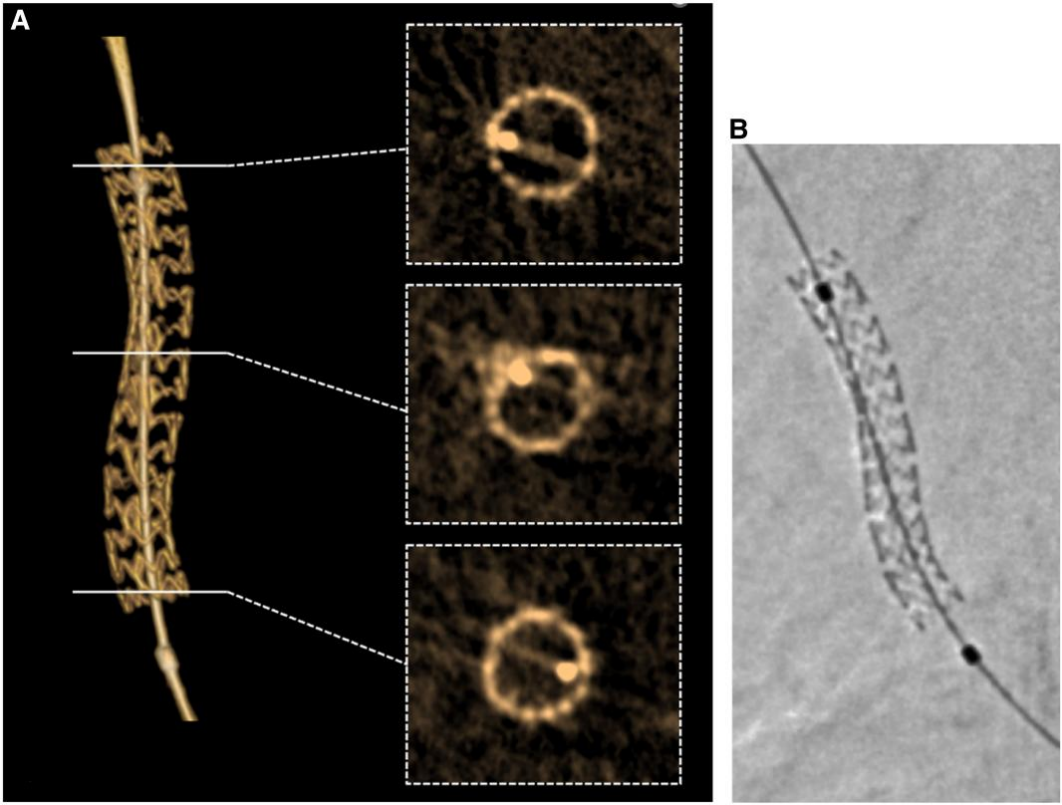
Example of a left circumflex artery. (*A*) 3D stent reconstruction (3DStent prototype). (*B*) Stent enhancement (StentViz). See movies attached for dynamic review of 3DStent 3D rendering view (see Supplementary data online, *Movie S2*) and 3DStent cross-sections views (see Supplementary data online, *Movie S3*). The corresponding 3D rotational angiography is available in Supplementary data online, *Movie S1*.

**Figure 3:**
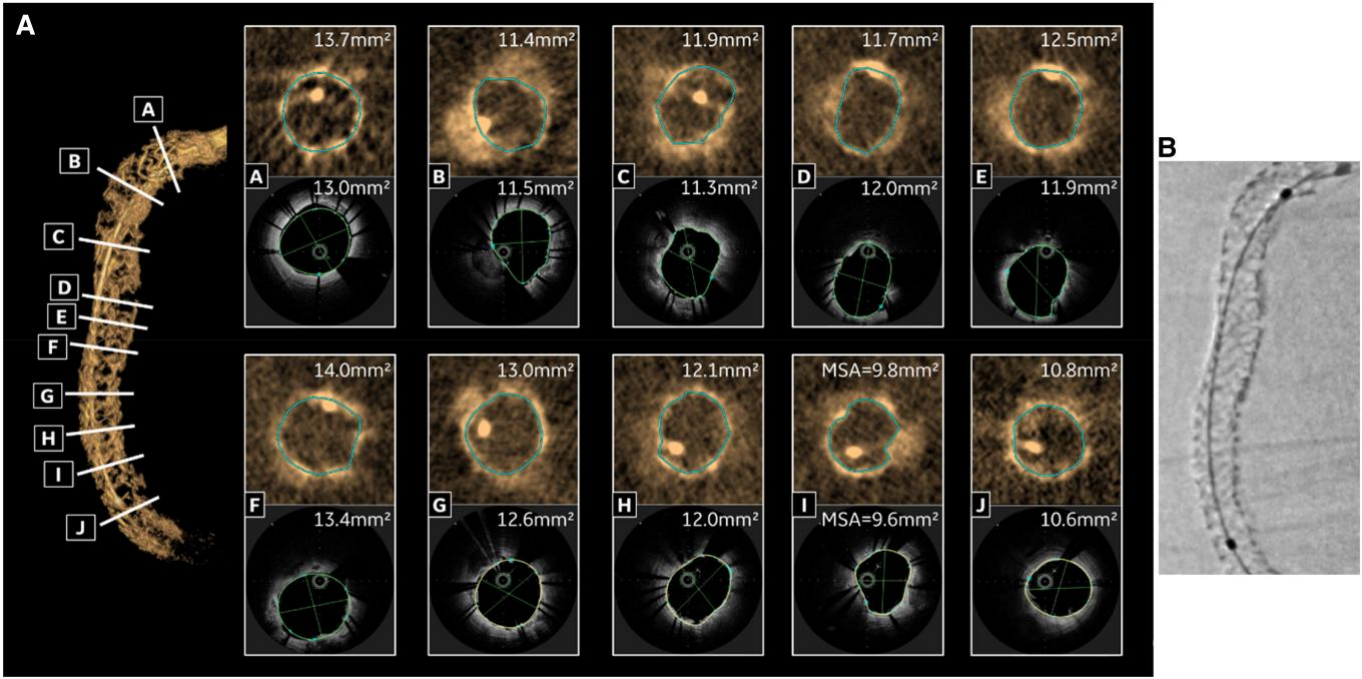
Example of a calcified RCA. (*A*) Stent cross-sections comparison between 3D stent reconstruction (3DStent prototype) and intra-vascular imaging. (*B*) Stent enhancement (StentViz). RCA, right coronary artery.

*Figures 2* and *3* illustrate two cases, showing the VR of the 3D image and the cross-sectional views of the reconstructed stent. The StentViz images are provided for comparison.

### Comparison with intra-vascular imaging in one case

**Figure 4:**
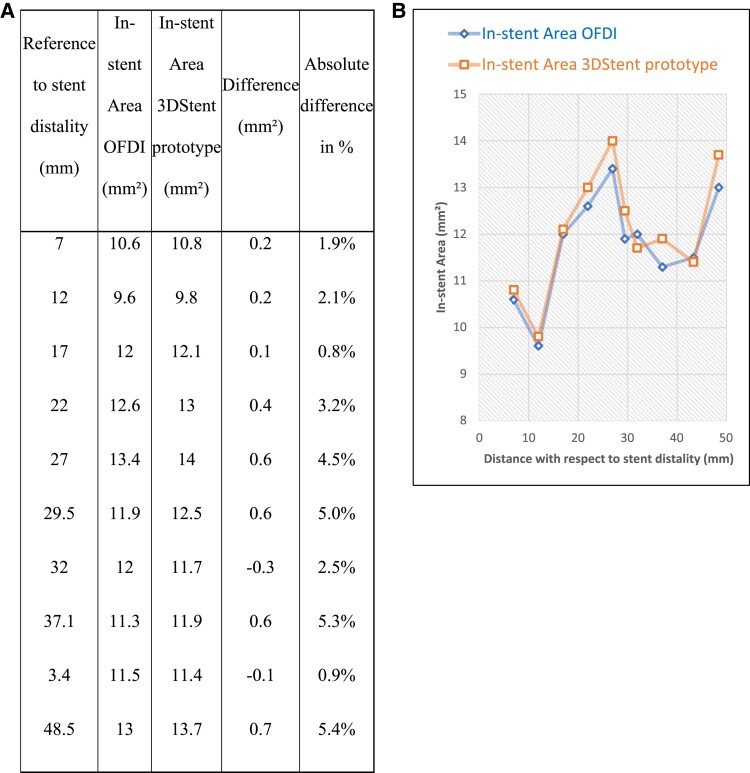
Comparative measurements of stent area with 3DStent prototype and intra-vascular imaging (OFDI) for the case illustrated in *Figure 3*. (*A*) Table with numerical values of in-stent area with 3DStent and OFDI. (*B*) Graphical curves of in-stent area with 3DStent and OFDI. OFDI, optical frequency domain imaging.

For the RCA case illustrated in *Figure 3*, an intra-vascular imaging was performed just before the 3D rotational acquisition, using optical coherence tomography with the Lunawave-OFDI Optical device (Terumo, Europe). The corresponding cross-sections between 3DStent prototype and intra-vascular imaging are shown in *Figure 3* and the in-stent area measurements are plotted in *Figure 4*. A good agreement is observed between the two technologies, both in terms of image content and quantitative measurements, with an average error of 3.2% and a maximum error of 5.4%.

### Image quality assessment and inter-observer agreement

**Figure 5:**
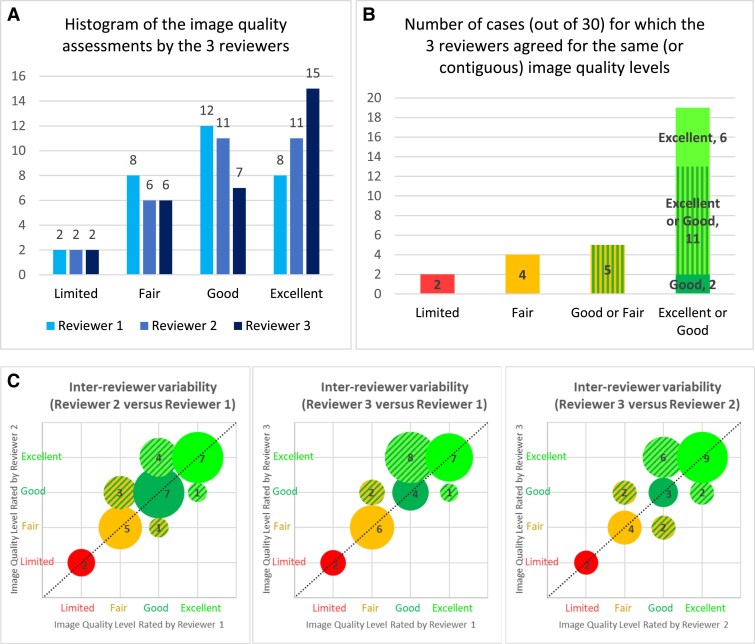
Result of the image quality assessments of the 3D stent reconstructions done by three different reviewers. (*A*) Histogram of the image quality levels attributed by each reviewer. (*B*) Agreement in the image quality levels between the three reviewers. (*C*) Image quality notes represented by pairs of reviewers.

Graph A of *Figure 5* represents the image quality assessment performed by three experienced interventional cardiologists during separate image reviews of the 3DStent prototype reconstructions. Overall, the quality of the 3DStent prototype reconstruction was evaluated as Excellent for 37.8% (34/3 × 30) of the cases, Good for 33.3% (30/3 × 30), Fair for 22.2% (20/3 × 30), and Limited for 6.7% (6/3 × 30).

The agreement among the three reviewers was deemed good, as illustrated in Graph B of *Figure 5*. They all agreed on the classification of 19/30 cases as either Excellent or Good (including 6 cases with full agreement for Excellent and 2 cases with full agreement for Good), 5/30 cases as either Good or Fair, 4/30 cases with a full agreement for Fair, and 2/30 cases with full agreement for Limited.

The agreement for each pair of reviewers was assessed using weighted Cohen’s kappa. Substantial agreement was found among reviewers 1 and 3 (0.80), and among reviewers 2 and 3 (0.78), while near-perfect agreement was found among reviewers 1 and 2 (0.82). Ratings by pairs of reviewers are illustrated in Graph C of *Figure 5*. All disagreements concern image quality notes belonging to contiguous categories.

The substantial to near-perfect agreement observed between the three reviewers justifies the assignment of a mean quality score to each of the 30 samples for the calculation of the Spearman coefficient, as proposed in the methodology section. A strong and significant correlation was found between the 3D mean quality score and the BMI (*ρ*=0.74, *P*-value < 0.001). Regarding the stent orientation, a weak and non-significant correlation with the 3D mean quality score was observed (*ρ*=0.34, *P*-value = 0.070).

### Quantitative comparison with 2D stent enhancement

In 12 out of 30 cases where StentViz was available, the clinical value of the 3D reconstruction of the stent was assessed as superior to StentViz in 9/12 cases by reviewer 1, in 10/12 cases by reviewer 2, and in 8/12 cases by reviewer 3. It was deemed equivalent to StentViz in the remaining cases, and was not assessed as inferior to StentViz in any case by any of the reviewers. Specifically, in respectively 4/12 cases (reviewer 1), 6/12 cases (reviewer 2) and 1/12 cases (reviewer 3), the 3D stent reconstruction identified a stent expansion defect that had not been detected with StentViz, or, conversely, it allowed for the confident conclusion that some doubts raised when looking at StentViz were not stent expansion defects. In the remaining cases where the clinical value of the 3D stent reconstruction was ranked superior to StentViz (5/12, 4/12, and 7/12 for reviewers 1, 2, 3, respectively), the reasons noted by the reviewers were that the 3D reconstruction is more comprehensive than 2D stent enhancement, particularly with a better understanding of calcium burden distribution, and more precise, with the capability to quantify defects in cross-sections as done in intra-vascular imaging, thus leading to a higher level of confidence in the clinical assessment.

### Dose

The median doses of the 3D rotational acquisitions for the 30 patients of the study are reported in *Table 3*, in terms of dose-area product (DAP) and cumulated air kerma (CAK). The median DAP for the 30 patients is 16.6 Gy.cm², equivalent to an effective dose of 3.32 mSv with a conversion factor of 0.2.^7^ Most 3D acquisitions were performed with no or very limited collimation in the image, therefore *Table 3* also reports the median value of the estimated DAP computed retrospectively by considering reasonable top-to-bottom collimation as recommended per 3DStent guidelines, considering for each case the length of the balloon and its orientation in the image. With this consideration, the estimated median DAP value would drop to 6.3 Gy.cm², equivalent to an effective dose of 1.26 mSv. These numbers must be compared with the typical dose of a PCI, with a median DAP of 30.7 Gy.cm² (equivalent to an effective dose of 6.8 mSv).^8^ Note that the numbers reported in *Table 3* correspond to cases acquired with the highest dose acquisition setting available for the 3DStent acquisition. A dose reduction by a factor of 2 could be achieved by using the lowest dose acquisition setting of 3DStent mode. As for the CAK, it is, by definition, not impacted by the collimation.

## Discussion

### Clinical context

This imaging modality is applied in the context of PCI. Studies^4^ have shown the importance of adequate stent expansion in reducing the risk of major adverse cardiovascular events (MACE). Stent enhancement^2^ is widely used to assess intra-procedural stent deployment; however, it only provides a single plane view of the stent. Intra-vascular imaging is a reference tool for assessing stent deployment but remains under-used for multiple reasons including additional costs and complexity. IVUS (and OCT) were used solely in 8.67% (and 0.6%) of PCI performed in the USA in 2019,^9^ respectively.

### Clinical workflow and image interpretation

The acquisition workflow requires some attention from the operator to adjust the table position with the deflated balloon in place. The stent does not need to be at the isocenter of the system, it only needs to remain in the field of view throughout the entire rotation. Once the acquisition setup is done, the operator may choose to limit their own radiation exposure by moving to a greater distance from the imaging system or going to the control room during the rotational acquisition. No dye injection is used during the 3D acquisition, which reduces the need to stay close to the patient.

The 3DStent volume can be assessed directly from tableside. As with any 3D imaging modality, it is convenient to be able to rotate and scale the 3D view. The most useful views are the reformatted MPR views, with a preference for the axial view, which lies in a plane perpendicular to the axis defined by the two markers of the balloon. Unlike intra-vascular imaging, it is possible to tilt the visualization plane to always keep it perpendicular to the stent axis, especially when the stent is curved.

During the image reviews, additional findings were identified with opacities visible at different locations. In some cases, they appear in the immediate vicinity of an under deployed section of a stent. These can be calcifications that prevented the proper deployment of the stent, as shown for the RCA case when looking at the corresponding slices in the intra-vascular imaging (see *Figure 3*).

### Impact on daily practice

Stent expansion is a well-known predictor of post-PCI MACE and intra-vascular imaging has demonstrated the importance of using high-pressure non-compliant balloons for post-dilation. 2D stent enhancement is commonly used to guide post-dilation but remains limited to a 2D view. With CMCT technology, 3D rotational angiography allows imaging a coronary stent in 3D in the cathlab without any additional device and may play a role in stent optimization as demonstrated in further studies.

3D rotational angiography is a well-known technique that should be applicable to most modern cathlabs. It is widely used in other clinical fields such as interventional neuroradiology. The reconstruction of the 3DStent relies on the proprietary CMCT technology, which is now on the market. This approach does not require additional expenses per case, unlike intra-vascular imaging. After adequate training of the clinical team, the entire workflow from acquisition to 3D volume assessment can be executed in a similar or shorter timeframe than intra-vascular imaging based on initial practical observations.

### Limitations

This is a single centre study with a limited number of cases. The acquisition workflow is slightly different from the final 3DStent product design. The main differences are related to user interface and exposure management. The 3D reconstructions were reviewed offline after the procedure.

The qualitative comparison with 2D stent enhancement was possible only in a subset of cases since 2D stent enhancement was not performed systematically at the exact same step of the procedure. It is available only for 12 of 30 cases.

From a clinical standpoint, this study is not sized to cover in depth the different variability in patient conditions such as BMI, stent location, length, diameter, and brand. It does not cover the variability associated with patient physiological conditions, the prevalence of calcifications, etc. Systematic additional studies will be necessary.

This study did not systematically include additional imaging with intra-vascular imaging, IVUS or OCT. Both are recognized gold standards for assessing stent under-expansion. Further studies would be helpful for assessing 3DStent in comparison to intra-vascular imaging.

In the absence of other imaging modalities, the assessment of the result is solely qualitative with a visual assessment by experienced cardiologists. This methodology is known to suffer from significant variability. However, it is an essential first step since it enables to confirm that the image content can be interpreted. The comparison with other imaging modalities shall come later to validate these findings.

## Conclusions

This first-in-man study has demonstrated that 3D rotational angiography is feasible during PCI procedures and can be used to reconstruct coronary stents in 3D in the cathlab using CMCT technology.

The ability to obtain quantitative measurements for the diameter and stent area has also been demonstrated, and for the case having intra-vascular imaging done, a good agreement was observed between the two sets of measurements.

The 3D reconstruction was evaluated as excellent or good in most patients by three interventional cardiologists in separate reviews, with substantial to near-perfect agreement among the ratings. Compared with 2D stent enhancement, 3D stent reconstruction could bring new insights and more confidence in the assessment of stent expansion. With no need for any intra-vascular imaging device, this CMCT technology is a promising tool to visualize the stent’s cross-sections from tableside during the procedure.

This study had a limited number of cases. Additional studies with the recently introduced 3DStent product are necessary to further assess this technology, validate the measurements that can be done on these 3D stent reconstructions (e.g. in-stent area) with respect to intra-vascular imaging, and evaluate its role in stent optimization during PCI.

### Ethics

The study protocol has been approved by the French Agency for the Safety of Health Products (ANSM registration no. ID RCB 2017-A00983–50) and received a favourable opinion by a French ethics committee (CPP Ile-de-France II). Patient data were de-identified to protect the identities of individuals. The participants gave written informed consent.

## Supplementary Material

qyaf065_Supplementary_Data

## Data Availability

For confidentiality and intellectual property reasons, the de-identified image data sets underlying this article are safeguarded by ICV GVM La Roseraie and GE HealthCare and cannot be made available to external parties. The comparative numerical measurements between 3DStent and OFDI are available in the article for the case having received both imaging modalities. The data underlying the qualitative analysis of the images by the three reviewers will be shared upon reasonable request to the corresponding author.

## Lead author biography

Hakim Benamer graduated from the Medical School Pitié-Salpétrière, University Paris VI in 1987. Hakim Benamer obtained his Diploma of specialized studies in cardiovascular pathology in 1992. That same year, he then completed a Post Graduate Academic Degree in physiology and cardio-circulatory physiopathology with Dr Swynghedauw at Broussais Hospital. From 1997 to 2006, Hakim Benamer was Head of the Haemodynamics and Cardiac Catheterisation Laboratory at the Hôpital Européen de la Roseraie (Aubervilliers, France). He was then Interventional Cardiologist at the Hôpital Foch (Suresnes, France), and Head of cardiology department. Since 2008, he is an Interventional Cardiologist at the Institut Hospitalier Jacques Cartier (ICPS, Massy, France). Dr. Hakim Benamer is very interested in clinical research and has actively participated in a lot of clinical studies dedicated to interventional cardiology (devices, pharmacology, biology, diagnostic, and therapeutic strategies). He is Associate Member of the French Society of Cardiology (since January 1996) and Member of the Angiography and Interventional Cardiology working group of the French Society of Cardiology (1998). In 2022 and 2023, he was President of the GACI (Groupe Athérome coronaire et Cardiologie Interventionnelle), the community of the French Society of Cardiology (SFC) which aims to promote the scientific development of French interventional cardiology, and he was Past President in 2024 and 2025. Dr. Hakim Benamer has actively participated in the development of the Radial course on PCR edu online.
