# Supplementary material for: First-in-man study of coronary stent 3D reconstruction in the cathlab using rotational angiography
Source: Eur Heart J Imaging Methods Pract. 2025 May 28;3(2):qyaf065. doi: 10.1093/ehjimp/qyaf065 (PMC12412210; doi:10.1093/ehjimp/qyaf065)
Supplement: qyaf065_Supplementary_Data [file qyaf065_supplementary_data.zip › Supplementary Movie Legends.docx]

# **Supplementary data**

**Movie 1** – Example of rotational angiography for 3D stent reconstruction.

**Movie 2** – Volume rendering view of 3D stent reconstruction.

**Movie 3** – Cross-section view of 3D stent reconstruction.
